# Modified IMPROVE VTE Risk Score and Elevated D-Dimer Identify a High Venous Thromboembolism Risk in Acutely Ill Medical Population for Extended Thromboprophylaxis

**DOI:** 10.1055/s-0040-1705137

**Published:** 2020-03-13

**Authors:** Alex C. Spyropoulos, Concetta Lipardi, Jianfeng Xu, Colleen Peluso, Theodore E. Spiro, Yoriko De Sanctis, Elliot S. Barnathan, Gary E. Raskob

**Affiliations:** 1 The Donald and Barbara Zucker School of Medicine at Hofstra/Northwell, The Feinstein Institute for Medical Research and Department of Medicine, Anticoagulation and Clinical Thrombosis Services Northwell Health at Lenox Hill Hospital, New York, New York, United States; 2 Janssen Research & Development, LLC, Raritan, New Jersey, United States; 3 Thrombosis and Hematology Therapeutic Area, Clinical Development, Pharmaceuticals, Bayer U.S. LLC, Whippany, New Jersey, United States; 4 College of Public Health, The University of Oklahoma Health Sciences Center, Oklahoma City, Oklahoma, United States

**Keywords:** venous thromboembolism, medically ill, D-dimer, VTE risk score, extended thromboprophylaxis

## Abstract

An individualized approach to identify acutely ill medical patients at increased risk of venous thromboembolism (VTE) and a low risk of bleeding to optimize the benefit and risk of extended thromboprophylaxis (ET) is needed. The International Medical Prevention Registry on Venous Thromboembolism (IMPROVE) VTE risk score has undergone extensive external validation in medically ill patients for in-hospital use and a modified model was used in the MARINER trial of ET also incorporating an elevated D-dimer. The MAGELLAN study demonstrated efficacy with rivaroxaban but had excess bleeding. This retrospective analysis investigated whether the modified IMPROVE VTE model with an elevated D-dimer could identify a high VTE risk subgroup of patients for ET from a subpopulation of the MAGELLAN study, which was previously identified as having a lower risk of bleeding. We incorporated the modified IMPROVE VTE score using a cutoff score of 4 or more or 2 and 3 with an elevated D-dimer (>2 times the upper limit of normal) to the MAGELLAN subpopulation. In total, 56% of the patients met the high-risk criteria. In the placebo group, the total VTE event rate at Day 35 was 7.94% in the high-risk group and 2.83% for patients in the lower-risk group. A reduction in VTE was observed with rivaroxaban in the high-risk group (relative risk [RR]: 0.68, 95% confidence interval [CI]: 0.51–0.91, *p* = 0.008) and in the lower-risk group (RR: 0.69, 95% CI: 0.40 -1.20, *p* = 0.187). The modified IMPROVE VTE score with an elevated D-dimer identified a nearly threefold higher VTE risk subpopulation of patients where a significant benefit exists for ET using rivaroxaban.

## Introduction

The identification of hospitalized medically ill patients at high risk of venous thromboembolism (VTE) with a low risk of bleeding that would benefit from extended thromboprophylaxis (ET) in the post-hospital discharge period remains an important unmet clinical need. 1 It is estimated that approximately one-quarter of hospitalized medically ill patients are at high VTE risk. 1 2 Data from randomized controlled trials of ET with the direct oral anticoagulants (DOACs) betrixaban and rivaroxaban reveal net clinical benefit of such a strategy in key patient groups or subgroups, and these agents have gained regulatory approval in the US. 3 4 However, identification of such patients using a standardized approach remains difficult.

Antithrombotic guidelines have suggested or recommended against routine use of ET in medically ill patients but have adopted an individualized approach in assessing a hospitalized medical patient's risk of VTE and bleeding. 5 6 The International Medical Prevention Registry on Venous Thromboembolism (IMPROVE) tool is a weighted VTE risk score that has been derived specifically in medically ill patients and has undergone extensive external validation. 7 8 9 10 A modified version of the IMPROVE VTE risk score that incorporated an elevated D-dimer also was used as part of the inclusion criteria for the MARINER trial of ET with rivaroxaban. 11 More recently, the IMPROVE VTE risk score has been highlighted as a key VTE risk assessment model for clinician decision-making in assessing VTE risk in hospitalized medical patients. 6

The results of the MAGELLAN study of ET in hospitalized medically-ill patients demonstrated superior efficacy with rivaroxaban compared with standard duration enoxaparin but had excess bleeding. 12 The study included a list of additional VTE risk factors as part of the inclusionary criteria to enrich the population for VTE events. 13 We, therefore, conducted this retrospective analysis to investigate whether the modified IMPROVE VTE model with an elevated D-dimer could identify a high VTE risk subgroup of patients for ET from a subpopulation of the MAGELLAN study, which was previously identified as having a lower risk of bleeding. 4

## Materials and Methods

### Study Design

The MAGELLAN protocol and results have been reported previously. 12 13 Briefly, the MAGELLAN study [NCT00571649] was a multicenter, randomized, double-blind, parallel-group efficacy, and safety study comparing rivaroxaban (10 mg once daily) administered for 35 ± 4 days to enoxaparin (40 mg once daily) administered for 10 ± 4 days followed by placebo, for the prevention of VTE in hospitalized acutely ill medical patients during the in-hospital and post-hospital discharge period. Eligible patients included adults who were at least 40 years of age, hospitalized for an acute medical illness (i.e., heart failure, active cancer, acute ischemic stroke, acute infectious and inflammatory disease, and acute respiratory insufficiency), at risk of VTE due to moderate or severe immobility, and had additional risk factors for VTE such as prolonged immobilization, age ≥75 years, history of cancer, history of VTE, history of heart failure, thrombophilia, acute infectious disease contributing to the hospitalization, and body mass index ≥35 kg/m ^2^ .

The MAGELLAN subpopulation, 4 which was not prespecified as a part of the original trial methodology, consisted of patients in the MAGELLAN trial without any of the following five risk factors for International Society on Thrombosis and Haemostasis (ISTH) defined major bleeding: active cancer at randomization, dual antiplatelet therapy at baseline, a medical history of bronchiectasis/pulmonary cavitation, active gastroduodenal ulcer, or any bleeding in the previous 3 months prior to randomization. Patients who met one or more of these criteria prior to or at randomization were excluded from the MAGELLAN subpopulation used in this analysis. These five exclusion criteria were part of the MARINER study [NCT02111564] that tested ET with rivaroxaban in the post-hospital discharge period. The MARINER protocol and results have been reported previously. 11 14

In this retrospective analysis, using the MAGELLAN subpopulation, eligible patients also had to have additional risk factors for venous thromboembolism as used by the inclusion criteria of the MARINER trial, 11 namely by a total modified IMPROVE risk score of 4 or higher (scores range from 0 to 10, with higher scores indicating a higher risk of venous thromboembolism; minimal clinically important difference, 2) or a risk score of 2 or 3 plus a plasma D-dimer level at the time of screening of more than twice the upper limit of the normal range. D-dimer was measured centrally using the assay STA Liatest D-DI (Diagnostica Stago S.A.S., Asnières sur Seine, France) that has an upper limit of normal of 0.5 mg/mL. The modified IMPROVE VTE risk score was calculated from available information on the patient’s risk factors for thromboembolism in the original case report forms of the MAGELLAN database ( Table 1 ). We used the modified IMPROVE VTE risk score, which included patients with a history of cancer in remission excluding nonmelanoma skin cancer present at any time in the past 5 years, as well as complete immobilization as defined by the MAGELLAN study for at least 1 day (as opposed to at least 7 days). 11

**Table 1:** Modified IMPROVE VTE risk score 11

VTE risk factor	VTE risk score
Previous VTE	3
Known thrombophilia a	2
Current lower limb paralysis or paresis b	2
History of cancer c	2
ICU/CCU stay	1
Complete immobilization d ≥ 1 d	1
Age ≥60 y	1

Abbreviations: CCU, cardiac care unit; ICU, intensive care unit; IMPROVE, International Medical Prevention Registry on Venous Thromboembolism; NIH, National Institutes of Health; VTE, venous thromboembolism.

a A congenital or acquired condition leading to excess risk of thrombosis (e.g., factor V Leiden, lupus anticoagulant, factor C or factor S deficiency).

b Leg falls to bed by 5 seconds, but has some effort against gravity (taken from NIH stroke scale).

c Cancer (excluding nonmelanoma skin cancer) present at any time in the past 5 years (cancer must be in remission to meet eligibility criteria).

d Immobilization is being confined to bed or chair with or without bathroom privileges.

### Study Outcomes

The efficacy and safety outcomes assessed in this analysis were performed as specified in the original MAGELLAN trial. Briefly, the primary efficacy outcome was the composite of asymptomatic proximal deep vein thrombosis (DVT) in lower extremity detected by mandatory bilateral lower extremity venous ultrasonography, symptomatic DVT in lower extremity, proximal or distal, symptomatic, nonfatal pulmonary embolism (PE), and VTE-related death (defined as either a well-documented fatal PE, or sudden death with no other plausible explanation). In this analysis, the primary population for the evaluation of superiority testing was the modified intent to treat (mITT) population at Day 35. The mITT population included patients who were valid for the safety analysis with an adequate ultrasonography assessment of VTE at Day 35. The events were assessed by an independent UAC (Ultrasonography Adjudication Committee) and the CEAC (Clinical Events Adjudication Committee). The principal safety outcome was the incidence of treatment-emergent clinically relevant bleeding defined as the composite of treatment-emergent major bleeding using ISTH definitions and nonmajor clinically relevant (NMCR) bleeding at Day 35. 13 This endpoint was assessed from the first dose of study drug until the end of treatment plus 2 days.

### Statistical Analyses

The relative risk (RR) for the primary efficacy outcome was calculated to evaluate the superiority of rivaroxaban over enoxaparin/placebo in mITT population at Day 35 and the RR for the principal safety outcome was provided in the safety population. The 95% confidence interval's (CI) and two-sided *p* -values were calculated using the Mantel-Haenszel method, which is available in PROC FREQ of SAS 9.4. 15 If not specified otherwise, region was the only stratification factor in our calculation.

## Results

### Baseline Patient Characteristics

**Fig. 1:**
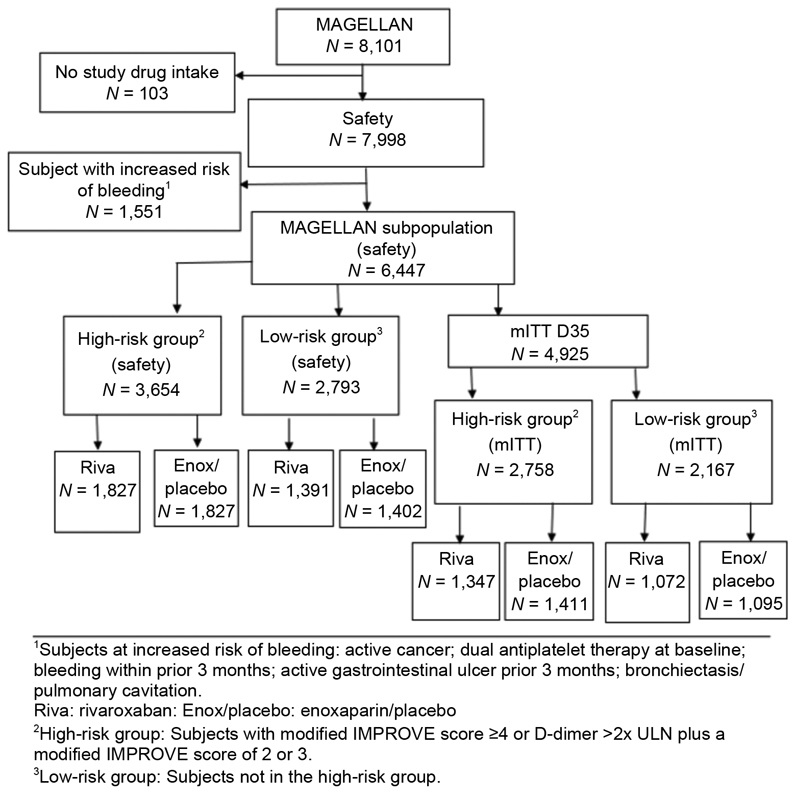
Subject disposition. mITT, modified intent to treat; ULN, upper limit of normal.

In the safety population, a total of 3,654 patients met the modified IMPROVE and elevated D-dimer criteria (i.e., high-risk group), while 2,793 patients did not meet the modified IMPROVE VTE risk score and elevated D-dimer criteria (i.e., low-risk group) ( Fig. 1 ). The demographics were generally balanced between treatment groups in both risk groups ( Table 2 ). In general, the patients in the high-risk group were older, with more renal impairment, and more often hospitalized for acute ischemic stroke. The prevalence of each clinical risk factor as used in the modified IMPROVE score was substantially lower in the low-risk group ( Table 2 ).

**Table 2:** Key baseline demographics (MAGELLAN subpopulation: safety analysis set)

	High-risk group		Low-risk group	
	Rivaroxaban *N* = 1,827	Enoxaparin/placebo *N* = 1,827	Rivaroxaban *N* = 1,391	Enoxaparin/placebo *N* = 1,402
Age (mean)	73.7	73.6	64.2	64.3
Age ≥75 ( *n* /%)	918 (50.2)	927 (50.7)	363 (26.1)	367 (26.2)
Male sex ( *n* /%)	947 (51.8)	901 (49.3)	794 (57.1)	760 (54.2)
White race ( *n* /%)	1273 (69.7)	1284 (70.3)	989 (71.1)	961 (68.5)
Weight (mean)	75.4	75.3	83.1	82.2
BMI (mean)	27.7	27.6	30.0	29.9
Baseline creatinine clearance ( *n* /%)				
< 30	54 (3.0)	40 (2.2)	13 (0.9)	11 (0.8)
30 to < 50	447 (24.5)	468 (25.6)	190 (13.7)	194 (13.8)
≥ 50	1298 (71.0)	1288 (70.5)	1158 (83.2)	1174 (83.7)
Missing	28 (1.5)	31 (1.7)	30 (2.2)	23 (1.6)
Reason for hospitalization ( *n* /%)				
Heart failure	568 (31.1)	598 (32.7)	536 (38.5)	531 (37.9)
Acute ischemic stroke	466 (25.5)	483 (26.4)	119 (8.6)	102 (7.3)
Acute infectious disease	838 (45.9)	793 (43.4)	728 (52.3)	742 (52.9)
Inflammatory disease	66 (3.6)	62 (3.4)	65 (4.7)	65 (4.6)
Acute respiratory insufficiency	458 (25.1)	472 (25.8)	460 (33.1)	478 (34.1)
History of VTE ( *n* /%)	165 (9.0)	151 (8.3)	0	0
History of cancer ( *n* /%)	333 (18.2)	306 (16.7)	19 (1.4)	22 (1.6)
ICU or CCU stay ( *n* /%)	233 (12.8)	235 (12.9)	22 (1.6)	25 (1.8)
Current lower-limb paralysis or paresis ( *n* /%)	430 (23.5)	459 (25.1)	119 (8.6)	100 (7.1)
D-dimer level more than 2x ULN ( *n* /%)	1268 (69.5)	1298 (71.0)	156 (11.2)	166 (11.8)
Modified IMPROVE score ( *n* /%)				
< 2	0	0	445 (32.0)	452 (32.2)
2	764 (41.8)	782 (42.8)	784 (56.4)	801 (57.1)
3	55 (3.0)	64 (3.5)	162 (11.6)	149 (10.6)
≥ 4	1008 (55.2)	981 (53.7)	0	0

Abbreviations: BMI, body mass index; CCU, cardiac care unit; DVT, deep vein thrombosis; ICU, intensive care unit; IMPROVE, International Medical Prevention Registry on Venous Thromboembolism; ULN, upper limit of normal; VTE, venous thromboembolism.

### Efficacy Outcomes

Among the high-risk group, the incidence of the primary efficacy outcome at Day 35 (mITT analysis set) was 5.42% (73/1347) in the rivaroxaban group and 7.94% (112/1411) in the enoxaparin/placebo group (RR: 0.68, 95% CI: 0.51–0.91, *p* = 0.008, absolute risk reduction [ARR] 2.52%) ( Table 3 ). In the low-risk group, the incidence in the rivaroxaban group was 1.96% (21/1072) and 2.83% (31/1095) in the enoxaparin/placebo group (RR: 0.69, 95% CI: 0.40–1.20, *p* = 0.187, ARR 0.87%). The predominant event in both risk groups was asymptomatic lower extremity proximal DVT. The results by component of the composite are given in Table 3 .

**Table 3:** Efficacy endpoints (MAGELLAN subpopulation: mITT analysis set, day 35)

	High-risk group				Low-risk group			
	Rivaroxaban *N* = 1,347 *n* (%)	Enoxaparin/placebo *N* = 1,411 *n* (%)	Relative risk (95% CI)	*p* -Value	Rivaroxaban *N* = 1,072 *n* (%)	Enoxaparin/placebo *N* = 1,095 *n* (%)	Relative risk (95% CI)	*p* -Value
Symptomatic nonfatal PE, symptomatic DVT, VTE-related death, asymptomatic proximal lower DVT	73(5.42)	112(7.94)	0.68 (0.51–0.91)	0.008	21(1.96)	31(2.83)	0.69 (0.40–1.20)	0.187
Symptomatic nonfatal PE	4(0.30)	8(0.57)	0.52 (0.16–1.71)	0.275	3(0.28)	2(0.18)	1.50 (0.24–9.32)	0.660
Symptomatic DVT	7(0.52)	9(0.64)	0.81 (0.31–2.16)	0.677	2(0.19)	1(0.09)	1.96 (0.19–20.07)	0.562
Asymptomatic lower proximal DVT	57(4.23)	87(6.17)	0.68 (0.49–0.95)	0.020	16(1.49)	23(2.10)	0.72 (0.38–1.35)	0.297
VTE-related death	12(0.89)	19(1.35)	0.67 (0.33–1.37)	0.266	3(0.28)	7(0.64)	0.43 (0.11–1.68)	0.210

Abbreviations: CI, confidence interval; DVT, deep vein thrombosis; mITT, modified intent to treat; PE, pulmonary embolism; VTE, venous thromboembolism.

### Safety Outcomes

In the high-risk group, the incidence of major bleeding through Day 35 was 0.82% (15/1827) in the rivaroxaban group and 0.66% (12/1827) in the enoxaparin/placebo group (RR: 1.24, 95% CI: 0.58–2.65, *p* = 0.571, absolute risk increase (ARI) 0.16%) ( Table 4 ). In the low-risk group, the incidence was 0.50% (7/1391) in the rivaroxaban group and 0.21% (3/1402) in the enoxaparin/placebo group (RR: 2.34, 95% CI: 0.60–9.04, *p* = 0.206, ARI 0.29%).

**Table 4:** Safety endpoints (MAGELLAN subpopulation: safety analysis set, treatment emergent events at day 35)

	High-risk group				Low-risk group			
	Rivaroxaban *N* = 1,827 *n* (%)	Enoxaparin/placebo *N* = 1,827 *n* (%)	Relative risk (95% CI)	*p* -Value	Rivaroxaban *N* = 1,391 *n* (%)	Enoxaparin/placebo *N* = 1,402 *n* (%)	Relative risk (95% CI)	*p* -Value
Clinically relevant bleeding	70 (3.83)	35 (1.92)	1.98 (1.33–2.96)	<0.001	44 (3.16)	14 (1.00)	3.20 (1.77–5.81)	<0.001
Major bleeding	15 (0.82)	12 (0.66)	1.24 (0.58–2.65)	0.571	7 (0.50)	3 (0.21)	2.34 (0.60–9.04)	0.206
Clinically relevant nonmajor bleeding	56 (3.07)	23 (1.26)	2.41 (1.49–3.90)	<0.001	37 (2.66)	11 (0.79)	3.44 (1.77–6.70)	<0.001

Abbreviations: CI, confidence interval; ISTH, International Society on Thrombosis and Haemostasis.

Note: Clinically relevant bleeding is a composite of major and nonmajor clinically relevant bleedings. Major and clinically relevant nonmajor bleeding were adjudicated per the ISTH definitions.

The incidence of NMCR bleeding was significantly higher with rivaroxaban treatment compared with enoxaparin/placebo in both risk groups. In the high-risk group, the incidence of NMCR bleeding was 3.07% (56/1827) in the rivaroxaban group versus 1.26% (23/1827) in the enoxaparin/placebo group (RR: 2.41, 95% CI: 1.49–3.90, *p* < 0.001, ARI 1.81%). In the low-risk group, the incidence in the rivaroxaban group was 2.66% (37/1391) versus 0.79% (11/1402) in the enoxaparin/placebo group (RR: 3.44, 95% CI: 1.77–6.70, *p* < 0.001, ARI 1.87%) ( Table 4 ).

## Discussion

This study revealed that a modified IMPROVE VTE risk score with a score of 4 or more or a score of 2 or 3 plus an elevated plasma D-dimer level is able to identify a subpopulation of hospitalized medically ill patients with (1) a nearly threefold higher VTE risk compared with the lower VTE risk group (total VTE 7.94 vs. 2.83%) and (2) in whom a significant 32% risk reduction with ET occurred. There were no statistically significant differences in both clinically relevant and major bleeding between the high and low VTE risk subpopulations, with major bleeding rates of <1.0%. Our analysis, therefore, identified a high VTE risk subpopulation of hospitalized medically ill patients, representing ∼57% of the study population, for whom there is a net clinical benefit using rivaroxaban for ET.

There continues to be an unmet medical need of how to best define a high VTE risk medically ill population that would benefit from ET as two DOACs, betrixaban and rivaroxaban are available for use with this indication in the US. Antithrombotic guidelines have moved away from group-based or universal definitions of VTE risk categories of medically ill patients to that of an individualized approach. 5 6 The advantages of using weighted and scored risk assessment models for VTE include the potential to allow for more tailored strategies for thromboprophylaxis and an improved estimation of the risk–benefit profile for pharmacologic thromboprophylaxis in a particular patient. 16 The most recent guidelines have highlighted the IMPROVE VTE risk score for clinician decision-making in assessing VTE risk in hospitalized medical patients, as it has gone through extensive external validation. 6 Both the original derivation study and external validation efforts have established a cutoff score of 4 or more as defining a high VTE risk population of medically ill patients having a VTE incidence of ∼ 4%, although these efforts included only in-hospital medically-ill patients. 7 8 Recent evidence has also established an elevated D-dimer using a cutoff of more than twice the upper limit of normal as an important novel biomarker in defining high VTE risk medically ill populations both in-hospital and in the post-hospital discharge period. 13 17 18 In addition, a version of the IMPROVE VTE risk score that incorporated elevated D-dimers with a score of 2 points improved model discrimination and identified a subpopulation (67%) of medically ill patients undergoing ET with betrixaban for whom a nearly threefold greater risk of VTE exists (hazard ratio: 2.73, 95% CI: 1.52–4.90, *p* < 0.001), thus suggesting that the IMPROVE VTE risk score plus elevated D-dimers can be used to define a population that would benefit from an extended post-hospital discharge course of thromboprophylaxis. 19 An identical version of the IMPROVE plus D-dimer tool used in our study, namely a modified IMPROVE VTE risk score with a score of 4 or more or a score of 2 or 3 plus an elevated plasma D-dimer level, was used prospectively in the large MARINER trial of ET with rivaroxaban, and identified an at-VTE risk population of medically ill patients with an incidence of symptomatic VTE and VTE-related death of 1.1% in the placebo group, although this was less than the expected 2.0 to 2.5% incidence. 11 14 The lower than expected symptomatic and fatal VTE incidence seen in the MARINER clinical trial compared with previous outcome studies was likely due to the fact that in-hospital VTE events were not included and the study's time period of 45 days for accruing events was less than the 90-day period from previous studies. 7 8

Our findings have important clinical implications. Clinical factors (advanced age, history of VTE, known thrombophilia, cancer, lower extremity immobility/paresis, intensive/coronary care unit stay) included in the IMPROVE VTE risk score and a biomarker (elevated D-dimer) have been independently associated with elevated VTE risk in hospitalized medical patients. 19 20 In addition, the inclusion criteria of the APEX study using both the key clinical criteria seen in our analysis (advanced age, history of VTE, immobility, and cancer) in addition to an elevated D-dimer identified a high VTE risk population that benefited from ET with betrixaban. 3 Our results suggest that by incorporating these individual factors in a weighted and scored VTE risk assessment model, we may further define a nearly threefold higher VTE risk subpopulation with sustained VTE risk up to 35 days after an index hospitalization. Importantly, there appeared to be no increased clinically relevant or major bleed risk in this high VTE risk subpopulation compared with the lower VTE risk group, provided that populations with key clinical risk factors for bleeding, namely active cancer, dual antiplatelet therapy at baseline, a medical history of bronchiectasis/pulmonary cavitation, active gastroduodenal ulcer, or any bleeding in the previous 3 months prior to hospitalization, are excluded from ET. Thus, our results would further define ∼60% of an at-VTE risk population of medically ill patents that would benefit from both in-hospital and ET, or ∼25 to 30% of the total population of medically ill patients. 1 2 The modified IMPROVE VTE risk score plus elevated D-dimer used in our study has the potential to be incorporated as a clinical decision support tool within a health system's electronic health records both at admission and at discharge, where previous efforts in hospitalized medical patients have shown an increase in uptake of appropriate thromboprophylaxis as well as a decrease in VTE using electronic or physician alerts at admission and discharge. 21 22

Limitations of the present analysis include its retrospective design in a subpopulation of the original MAGELLAN study population, which can introduce recall bias. However, individual risk factors included in the VTE risk score were collected prospectively in a standardized data collection form. Second, although D-dimer measurements can be influenced by the types of analytic methods and reporting standards of different laboratories, we used a centralized laboratory measurement to define the assay's upper limits of normal in prespecified time periods. Third, we included in our analysis the primary efficacy outcome of asymptomatic lower extremity DVT that was a key component of the original study's primary efficacy outcome, as clinical trial data now reveals a consistent and strong association of asymptomatic lower extremity DVT and mortality in medically ill patients. 23 24 This significant association with asymptomatic proximal DVT found by screening compression ultrasonography and higher all-cause mortality in medically ill patients was also recently established in the MAGELLAN trial. 25 Fourth, these data were derived in a large clinical trial of hospitalized medically ill patients and may be less generalizable in routine hospital settings, although the IMPROVE VTE risk score has now been externally validated in over 100,000 medically ill patients in various healthcare settings. 8 9 10

## Conclusion

For hospitalized medically ill patients, the modified IMPROVE VTE risk score incorporating an elevated D-dimer as a biomarker identified a nearly threefold higher VTE risk patient population for whom a significant benefit exists for ET of up to 35 days with rivaroxaban. This same higher VTE risk subpopulation did not exhibit an increase in either clinically relevant or major bleeding compared with the lower VTE risk group, potentially identifying a patient population of hospitalized medically ill patients for whom a net clinical benefit exists for extended out-of-hospital thromboprophylaxis. Further prospective impact analysis should be undertaken before this VTE risk score is widely implemented in routine clinical settings.
